# Volatile organic compounds in exhaled breath: Applications in cancer diagnosis and predicting treatment efficacy

**DOI:** 10.1016/j.cpt.2024.12.004

**Published:** 2025-01-03

**Authors:** Yilu Gao, Baoqing Chen, Xingyuan Cheng, Shiliang LiuD, Qiaoqiao Li, Mian Xi

**Affiliations:** a State Key Laboratory of Oncology in South China, Collaborative Innovation Centre for Cancer Medicine, Guangdong Esophageal Cancer Institute, United Laboratory of Frontier Radiotherapy Technology of Sun Yat-sen University & Chinese Academy of Sciences Ion Medical Technology Co., Guangzhou, Guangdong 510060, China; b Department of Radiation Oncology, Sun Yat-sen University Cancer Center, Guangzhou, Guangdong 510060, China

**Keywords:** Neoplasms, Volatile organic compounds, Diagnosis, Prognosis

## Abstract

Volatile organic compounds (VOCs) are carbon-based chemicals characterized by high vapor pressure and low boiling points under standard temperature and pressure conditions. VOCs are categorized as exogenous or endogenous, depending on their source. Endogenous VOCs are metabolic byproducts eliminated via respiration. These compounds serve as indicators of human metabolic activity, reflecting differences in tumors compared to normal cell metabolism and the body's response to tumors. Examination of exhaled breath provides a noninvasive approach for assessing metabolic status by comparing VOC levels. Consequently, VOCs are increasingly studied as novel biomarkers for cancer screening, diagnosis, and treatment efficacy prediction. This review outlines VOC production mechanisms, their presence in tumor types, detection methodologies, and their implications for tumor screening, diagnosis, and prognosis. Nonetheless, challenges remain in the utilization of VOCs for cancer diagnosis and predicting treatment outcomes. Furthermore, this review discusses unresolved issues requiring attention to improve malignant tumor assessment, providing insights into their diagnosis, treatment, and prognosis.

## Highlights

• Insight into sources and generation mechanisms of volatile organic compounds (VOCs).
• An overview of devices used for the detection of VOCs.
• A comprehensive review of VOCs' role in cancer diagnosis, treatment prognosis, and recurrence monitoring.

## Graphical abstract

**Figure:**
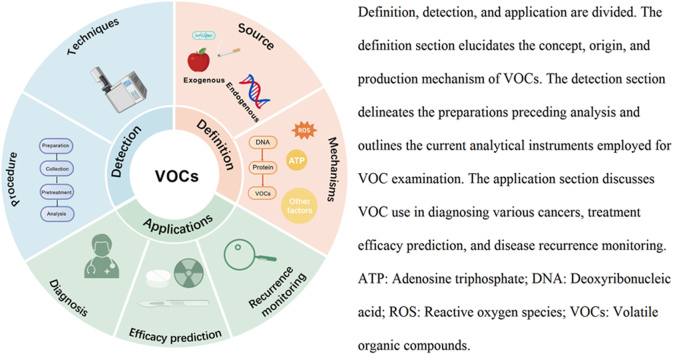

## Introduction

Malignant neoplasms are a major cause of global mortality. The World Health Organization reported nearly 20 million new cancer cases and approximately 9.7 million cancer-related deaths in 2022.^1^ Cancer management is complex, involving surgery, radiotherapy, chemotherapy, immunotherapy, targeted therapy, and other treatment strategies for different tumor types. Many cancers have a subtle onset and are often diagnosed at advanced local or metastatic stages, resulting in less-than-optimal treatment outcomes; therefore, early detection and diagnosis are crucial.

Currently, cancer screening and diagnosis rely on laboratory tests, imaging, endoscopic procedures, and histopathological biopsies. Although biomarkers, such as alpha-fetoprotein (AFP), prostate-specific antigen, and carcinoembryonic antigen, are used to diagnose and predict treatment responses in specific cancers, their overall sensitivity and specificity are limited. Most cancers lack distinct biomarkers for reliable diagnosis. Although exosomes and circulating tumor DNA hold promise for prediction, they are expensive and lack standardized detection protocols, hindering their widespread use. Therefore, innovative, noninvasive, user-friendly, and highly accurate biomarkers are urgently needed in clinical settings.

Human exhaled breath contains >1400 volatile organic compounds (VOCs), including hydrocarbons, oxygenated compounds, sulfur compounds, and nitrogen compounds.^2^ Pathological conditions can alter the VOCs produced in the body.^3^ Using precise analytical instruments enable the identification of unique VOC profiles changes in individuals with tumors. Detecting VOCs in exhaled breath shows promise for diagnosing malignant tumors.^4^, ^5^, ^6^, ^7^, ^8^, ^9^, ^10^ Although its use in predicting therapeutic efficacy is limited, it demonstrates considerable potential.^11^, ^12^, ^13^, ^14^ Studies have examined the predictive value of VOCs in assessing the therapeutic outcomes of chemotherapy, immunotherapy, targeted therapy, ablation, and transcatheter arterial chemoembolization (TACE). Analyzing VOC distribution before or during treatment can predict therapeutic efficacy variations, aiding physicians in adjusting treatment protocols and optimizing patient outcomes. This review explores recent developments in VOC detection for early screening, diagnosis, and treatment outcome prediction, aiming to provide insights into cancer diagnosis and management.

## Volatile organic compounds

### Definition of volatile organic compounds

According to the World Health Organization, VOCs are a diverse group of organic compounds characterized by a melting point below room temperature and pressure, and a boiling point falling between 50–260°C. Once generated, VOCs migrate to the lungs via the circulatory system and are eliminated via gas-blood exchange. Notably, VOCs are detectable in exhaled breath and are emitted through various bodily fluids such as skin secretions, urine, blood, saliva, and feces.^15^

VOCs can be categorized as exogenous or endogenous based on their source. Exogenous VOCs originate from external sources such as the environment, food, smoking, daily activities, drug metabolism, and microorganisms, whereas endogenous VOCs are intricately linked to metabolic processes within a person's tissues and cells.^16^ Moreover, in malignant tumors, research focuses primarily on alterations in endogenous VOCs. Tumor tissues exhibit substantial metabolic transformations compared to normal tissues.

**Figure 1:**
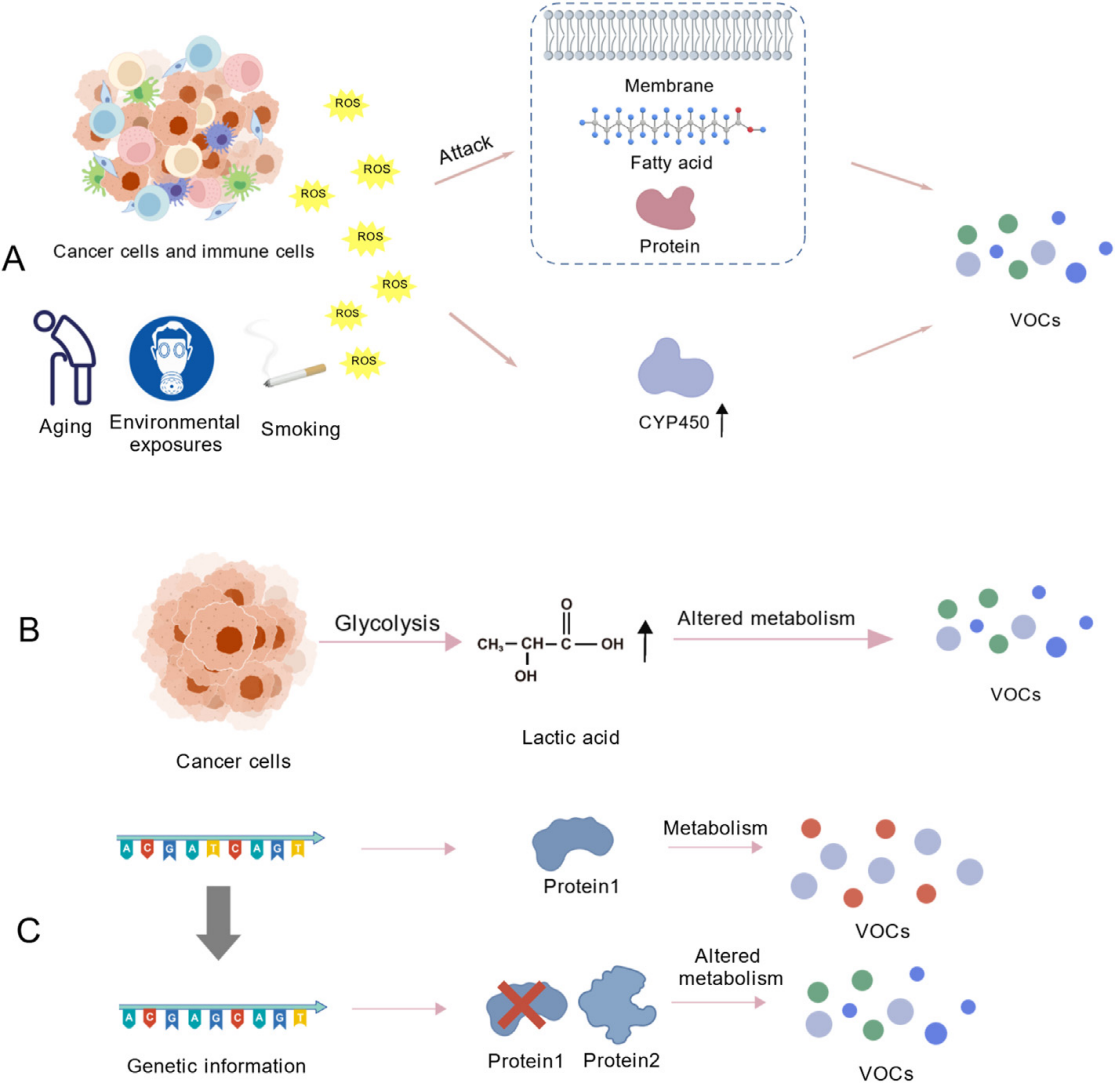
Mechanisms of VOC profile alterations in cancer. (A) Elevated oxidative stress in tumor cells triggers ROS generation. Tumor-induced inflammatory responses produce considerable ROS, damaging various biomacromolecules in the body, such as fatty acids and proteins, forming hydrocarbon substances. Elevated ROS levels in tumor tissues induce CYP450 overexpression, facilitating organic compound oxidation in the body, intricately linked to the synthesis of VOCs such as alcohols and aldehydes. Increased oxidative stress, induced by aging, environmental exposures, and smoking influences the VOC profile. (B) Hypoxic conditions in the tumor microenvironment prompt neoplastic cells to rely on glycolysis for energy, resulting in the accumulation of lactic acid. Alterations in metabolic pathways give rise to various intermediates, consequently influencing the profile and levels of VOCs. (C) Genomic instability and mutations, prominent characteristics of tumor cells, induce modifications in cellular signaling pathways or novel intermediate synthesis, leading to distinct VOC production. CYP450: Cytochrome P450; ROS: Reactive oxygen species; VOCs: Volatile organic compounds.

The precise pathological and physiological mechanisms underlying these VOC changes remain incompletely understood and are potentially associated with elevated oxidative stress and cytochrome P450 (CYP450) overexpression [Figure 1A],^3^ metabolic pathway alterations in [Figure 1B], genomic instability, and cancer cell mutations [Figure 1C].^3^^,^^17^ The shifts in the changes of VOCs in malignant tumors may originate from the tumor tissue or the inflammatory responses triggered by the tumor. Concurrently, the VOC profile is influenced by various factors, including the patient's disease status, comorbidities, medication use, and environmental factors.^18^^,^^19^ Notably, factors including aging, environmental exposures such as silica dust, and tobacco use may contribute to elevated oxidative stress.^20^, ^21^, ^22^ One study suggested that despite radical colorectal cancer (CRC) surgery, VOC distributions differ between patients with CRC and healthy individuals,^23^ indicating that VOC changes are influenced by the tumor and alterations in the tumor-induced microenvironment. Consequently, the analysis of VOCs holds promise for screening and diagnosing malignant tumors, predicting prognostic efficacy, and monitoring follow-up.

### Detection of volatile organic compounds

VOC analysis in exhaled breath is noninvasive, reducing patient discomfort. It allows convenient sampling with high reproducibility, enabling the collection of numerous samples within a short duration, and aiding tumor screening.

Prior to VOC analysis, gas collection is a prerequisite. Participants are typically instructed to fast, abstain from alcohol and smoking, and collect gas post-oral hygiene practices such as brushing teeth and rinsing the mouth. Tedlar bags are commonly used for collecting exhaled breath; despite their convenience, they are susceptible to contamination from ambient gases during storage and may be a source of contamination. Consequently, it is recommended to purge the bags with an inert gas before collection and simultaneously collect environmental gas samples to minimize errors. Following collection, gas analysis should be conducted promptly to lessen the impact of external gas interference. After collection, the gas undergoes pre-concentration, with adsorption tubes and solid-phase microextraction being the prevalent methods. Both techniques enrich VOCs in samples via adsorption, enhancing their concentration.^24^^,^^25^

The primary techniques for VOC detection include gas chromatography-mass spectrometry (GC-MS) and electronic noses. GC-MS is the standard method for VOC detection owing to its sensitivity to detect low VOC concentrations, conduct qualitative and quantitative analyses of exhaled breath, and identify unknown VOCs, aiding in the exploration of disease-specific VOCs.^26^ However, GC-MS is expensive, involves labor-intensive pretreatment steps, requires thermally stable compounds, and is limited to compounds with a molecular weight <1000 Da, restricting wider application.^27^ In contrast, an electronic nose is a device with an array of electronic chemical sensors and a pattern recognition system that can detect various odors by interacting with odor molecules,^28^ offering high sensitivity, portability, ease of use, and the ability to identify specific substances. In clinical settings, electronic noses for VOC detection efficiently provide a comprehensive distribution of relevant VOCs in a short time, enabling the development of differentiation models based on the VOC differences between patients with cancer and healthy individuals. However, an electronic nose cannot detect unknown VOCs and requires improved sensor stability.^29^

Selected ion flow tube mass spectrometry (SIFT-MS), proton transfer reaction-mass spectrometry (PTR-MS), and high-pressure photon ionization time-of-flight mass spectrometry (HPPI-TOFMS) are also available for VOCs detection. SIFT-MS offers real-time quantitative analysis, flexible sample handling, high sensitivity, specificity, and reproducibility; however, it is not suitable for trace gas or low-volatility compound analysis.^30^^,^^31^ PTR-MS enables real-time detection of VOC concentration without sample pretreatment and is unaffected by inorganic air components, with a sensitivity approximately two orders of magnitude higher than that of SIFT-MS. However, PTR-MS cannot differentiate between isomeric compounds and compounds with lower proton affinity than water molecules.^32^^,^^33^ HPPI-TOFMS facilitates high-throughput, real-time analysis without sample pretreatment, and is promising for complex gas analysis; however, it exhibits lower sensitivity to certain polar compounds, such as alcohols. The results are presented as mass-to-charge ratios (*m/z*), requiring further chemical analysis for precise compound identification.^34^

## Applications of volatile organic compounds in cancer screening and diagnosis

### Lung cancer

Recent studies highlight the potential utility of VOCs for detecting and characterizing malignant tumors. Wang et al^9^ analyzed exhaled breath samples from 84 patients with lung cancer (LC) before and after surgery as the discovery set, using HPPI-TOFMS. Sixteen VOCs were identified as potential biomarkers for LC from 28 previously reported LC-associated VOCs. A multivariable logistic regression model was constructed. The validation set consisted of 157 patients with LC and 368 healthy individuals. By incorporating 16 VOCs, the diagnostic model effectively differentiated between individuals with LC and healthy participants, yielding an area under the curve (AUC) of 0.95, sensitivity of 89.2%, specificity of 89.1%, and accuracy of 89.1%. This study integrated VOC biomarkers identified in prior research. Notable and distinct VOCs were selected from samples for model development and external validation to enhance generalizability. Nevertheless, HPPI-TOFMS, as a novel instrument, may lack reproducibility across diverse platforms.

Kort et al^35^ used an electronic nose to assess the exhaled breath of 575 participants, comprising 239 individuals with LC and 336 without. VOCs and clinical parameters were integrated into a multivariate logistic regression analysis. The model's diagnostic performance for LC was characterized by a sensitivity of 95%, specificity of 51%, negative predictive value of 94%, and AUC of 0.87 in the training dataset. In the validation dataset, the sensitivity remained at 95%, with a specificity of 49%, a negative predictive value of 94%, and an AUC of 0.86, suggesting that the model holds considerable promise for LC screening.

Binson et al^36^ examined exhaled breath samples from 114 patients with LC and 147 controls, analyzed them using an electronic nose, and constructed a model using the extreme gradient boosting (XGBoost) algorithm. The model demonstrated a sensitivity of 83.33%, specificity of 86.27%, and AUC of 0.87 for LC detection. Furthermore, the study explored the ability of VOCs to distinguish patients with LC at different clinical stages. The constructed model showed strong discrimination between stage I and stage IV patients (AUC of 0.82); however, weaker discrimination was noted between stage III and stage IV patients (AUC of 0.53).

Three studies examined the exhaled breath of individuals with and without LC using GC-MS, showing that the levels of 2-butanone and 1-propanol were considerably elevated in patients with LC compared to those in healthy individuals.^37^, ^38^, ^39^ Inhalation of 2-butanone from the external environment leads to its entry into the human body. Although current evidence does not indicate that 2-butanone is carcinogenic, it may facilitate cancer progression in conjunction with other substances, necessitating further investigation via animal studies.^40^ Moreover, increased concentrations of 2-butanone are possibly associated with smoking, as smokers typically exhibit higher levels than non-smokers.^37^ Conversely, 1-propanol, an alcohol, is primarily derived from dietary sources or alcoholic beverages and is also produced as a metabolic byproduct of P450 enzymes involved in hydrocarbon metabolism. Investigations into VOCs present in tumor tissues from patients with LC have revealed significantly elevated levels of both 2-butanone and 1-propanol, suggesting these compounds originate from tumor tissues.^38^ However, the precise mechanisms underlying the production of these two VOCs remain inadequately explored.

The model exhibited enhanced performance in identifying patients with LC compared to healthy controls (HCs); however, its diagnostic capabilities were lower in distinguishing patients with LC from individuals with other benign pulmonary conditions or different cancer subtypes.^41^, ^42^, ^43^ This discrepancy may stem from similarities in VOC profiles between benign pulmonary lesions and LC, and overlapping smoking behaviors among these patient groups may further contribute to analogous VOC profiles. Furthermore, participants’ lifestyles and other health conditions can influence VOC distributions. Consequently, when developing the model, accounting for these variables is crucial to minimize their confounding effects.

### Esophagogastric cancer

Markar et al^5^ used SIFT-MS for metabolic profiling of exhaled breath from 163 individuals with esophagogastric cancer (OGC) (72 cases of gastric cancer, 36 cases of gastroesophageal junction cancer, and 55 cases of esophageal cancer) and 172 controls. SIFT-MS identified five distinct compounds differing between the patient and control groups, leading to the development of a multivariable logistic regression model. The model exhibited an AUC of 0.85, demonstrating 80% sensitivity and 81% specificity for diagnosing OGC. However, the model could not differentiate between esophageal and gastric cancers. The multi-center design of this study enhanced the reliability of its findings. Most patients were in a locally advanced disease stage, necessitating further validation of the model for early diagnosis applicability.

Two studies identified higher concentrations of hexanoic acid, butanal, and decanal in patients with gastroesophageal cancer than in control groups,^5^^,^^44^ suggesting that the primary endogenous aldehyde source is lipid peroxidation, induced by oxidative stress. Healthy individuals maintain an oxidation–antioxidation equilibrium; however, cancer exacerbates oxidative stress, potentially increasing aldehyde levels.^44^ Detoxification, facilitated by aldehyde dehydrogenase (ALDH), serves as the principal mechanism for mitigating aldehyde accumulation, as ALDH further oxidizes aldehydes into carboxylic acids. Aldehyde accumulation in esophageal adenocarcinoma negatively correlates with aldehyde dehydrogenase 3 family member A2 (ALDH3A2) expression. In patients diagnosed with esophageal adenocarcinoma, ALDH3A2 expression was significantly reduced, contributing to aldehyde buildup. Moreover, diminished ALDH3A2 expressions correlate with a poorer prognosis in individuals with esophageal adenocarcinoma.^45^ Notably, carboxylic acids, products of aldehyde oxidation, are catalyzed by ALDH. ALDH activity increases in esophageal squamous cell carcinoma stem cells, which may be associated with upregulation of the heat shock protein 27 - protein kinase B - hexokinase 2 signaling pathway.^46^ Notably, ALDH isoenzyme expression varies across different cancers, with distinct ALDH subtypes exhibiting differential expression in tumor cells. Unlike ALDH3A2, aldehyde dehydrogenase 1 family member A1 (ALDH1A1), 1 family member B1 (ALDH1B1), and 3 family member A1 (ALDH3A1) expression levels are elevated in other malignant tumors and associated with poorer survival outcomes. A study leveraged RNA sequencing data from The Cancer Genome Atlas to assess the expression patterns of 19 ALDH isoforms across five prevalent human cancers. The isoforms aldehyde dehydrogenase 1 family member A2 (ALDH1A2), 2 family member (ALDH2), ALDH3A2, and 9 family member A1 (ALDH9A1) were downregulated in all cancers studied. In contrast, ALDH1B1, aldehyde dehydrogenase 1 family member L2 (ALDH1L2), and 18 family member A1 (ALDH18A1) are upregulated in most cancers. Furthermore, a subset of isoforms, including aldehyde dehydrogenase 1 family member L1 (ALDHA1L1), 3 family member B1 (ALDH3B1), 3 family member B2 (ALDH3B2), 4 family member A1 (ALDH4A1), and 7 family member A1 (ALDH7A1), show variable expression, being upregulated in certain cancers and downregulated in others.^47^ ALDH2 suppression leads to aldehyde accumulation; however, the specific functions of other ALDH isoforms are not well characterized.^48^ Consequently, the concentrations of aldehydes and carboxylic acids may differ across various cancer types. Understanding the expression patterns of ALDH isoenzymes in diverse cancers may provide valuable insights into the changes in aldehyde and carboxylic acid levels observed in patients with cancer.

### Hepatocellular carcinoma

Miller-Atkins et al^6^ conducted a study on exhaled breath samples from a cohort of healthy adults (*n* = 54) and patients with hepatocellular carcinoma (HCC) (*n* = 112). Using SIFT-MS, 17 VOCs that were significantly elevated in HCC samples were identified, with hydrogen sulfide levels higher in samples from the control group. A random forest machine learning approach was used to develop an algorithm model, which was assessed using leave-one-out cross-validation. This prediction model exhibited a sensitivity of 73% for diagnosing HCC, surpassing the sensitivity of AFP recorded at 53% within the same cohort. AFP has been extensively used in the screening and diagnosing HCC. However, its high false-negative rate highlights the need for diagnostic methods with increased sensitivity. Sukaram et al^14^ performed a comparative analysis of the diagnostic efficacy of acetone dimers and AFP in HCC. Acetone dimers demonstrated greater sensitivity (83.9%) in distinguishing HCC from non-HCC cases than AFP (62.4%).

Reprogramming of energy metabolism is a notable characteristic of cancer cells. Enhanced lipid metabolism provides increased energy for these cells. Acetone, a byproduct of fatty acid oxidation, is elevated in patients with cancer.^14^ However, factors such as physical exercise and dietary intake can influence these levels. Consequently, some studies have advised against the use of acetone as a reliable biomarker for cancer.^3^ Therefore, it is advisable to restrict food intake and exercise prior to the collection of exhaled breath samples, as this may help regulate acetone levels in patients and potentially improve assessment accuracy. Furthermore, empirical studies have demonstrated that hydrogen sulfide facilitates autophagy in HCC cells while suppressing their division, proliferation, and migratory capabilities.^49^ In this investigation exogenous hydrogen sulfide donor sodium hydrosulfide (NaHS) was administered to HCC cells, demonstrating that NaHS effectively downregulated phosphorylated-phosphatidylinositol 3-kinase (p-PI3K), phosphorylated-protein kinase B (p-Akt), and mammalian target of rapamycin (mTOR) expression in the PI3K/Akt/mTOR signaling pathway. Additionally, NaHS upregulates the expression of autophagy-related proteins, specifically microtubule-associated protein light chain 3-II (LC3-II) and autophagy-related protein 5 (Atg5), inhibiting division, proliferation, and migration of liver cancer cells.

### Breast cancer

Liu et al^10^ used HPPI-TOFMS to analyze the VOCs present in exhaled breath samples from 3175 individuals, comprising 2959 HCs and 216 patients with breast cancer (BC). Ten specific VOCs were identified as potential biomarkers. The random forest algorithm for model training yielded an AUC of 0.96 during internal validation. Subsequently, an additional cohort of 249 patients with BC and 1545 controls from three hospitals was recruited for external validation, achieving an AUC of 0.97 for the diagnostic model, an accuracy of 92.37%, and a sensitivity of 60.45% for BC detection. Including BC risk factors in the model improved its predictive performance. During external validation, the model achieved an AUC of 0.94, sensitivity of 89.16%, and specificity of 87.75% for BC diagnosis. Comparative analysis showed that HPPI-TOFMS outperformed GC-MS; however, it was less effective than the electronic nose. This multicenter study with a substantial sample size, successfully validated the generalizability of the model via external validation. Moreover, an investigation of exhaled breath profiles between patients with BC and controls highlighted hexanal as a compound exhibiting significant differences between the two cohorts in both studies.^50^^,^^51^ Hexanal, an aldehyde compound, is primarily a byproduct of ω-6 unsaturated fatty acid oxidation. In OGC, aldehydes are VOCs that result from lipid peroxidation.^50^ The electronic nose can detect specific VOCs, including hexanal levels, in exhaled breath of patients with cancer patients and control subjects, facilitating the assessment of hexanal concentration differences between these two groups.

These studies suggest that VOCs offer advantages for identifying and assessing cancerous tumors. However, numerous investigations are limited by small sample sizes and lack of external verification, necessitating large-scale studies. Furthermore, equipment discrepancies, sampling methodologies, and environmental factors may lead to variability in the outcomes. Factors such as medical conditions, smoking, diet, and gas collection bags can influence VOC analysis results. Smokers have higher levels of 2-butanone in their exhaled breath than non-smokers. Elevated concentrations of acetonitrile, benzene, toluene, ethylbenzene, and furane have also been reported in smokers.^52^^,^^53^ Furthermore, certain compounds are prevalent in the ambient environment, confounding detection outcomes.

Identical VOCs identified in the same cancer type across studies can serve as indicators of a particular cancer. Thus, confirming the diagnostic utility of these VOCs across various cancer types is essential. Variations in VOC compositions in different cancers suggest the potential for cancer categorization based on VOC profiles. Validation via cellular experiments is required to establish the cancer-specific characteristics of these VOCs. Notably, VOC type varies across different cancer types. Even within the same type of cancer, different detection devices may yield disparate results [Table 1]. However, the VOCs detailed in Table 1 originate from the exhaled breath of participants and are not necessarily endogenous. Factors such as smoking and environmental exposures complicate the identification of disease-specific VOC markers. For example, elevated levels of 2-butanone, acetonitrile, benzene, toluene, ethylbenzene, and furan have been detected in the exhaled breath of smokers. Limonene, a compound also detected in exhaled breath, is extensively used in food production and serves as an additive in cosmetics, industrial solvents, and pharmaceuticals.^54^ Furthermore, it has been observed that Tedlar bags can emit N,N-dimethyl acetamide, and phenol, potentially contaminating samples.^39^ Excluding VOC alterations from external factors during detection may improve the diagnostic accuracy of VOC analysis.

**Table 1:** Examples of changes in VOCs in different cancer types.

Cancer type	Study	Analytical platform	Groups	Change of VOC levels in patients		AUC	Sensitivity, %	Specificity, %	Accuracy, %
Lung	Peng et al^55^	GC-MS	HC *vs.* LC	1-Methyl-4-(1-methylethyl)benzene, dodecane	↓	NA	NA	NA	NA
				Toluene, 3,3-dimethyl pentane, 2,3,4-trimethyl hexane	↑				
	Wang et al^9^	HPPI-TOFMS	HC *vs.* LC	Acetaldehyde, 2-hydroxyacetaldehyde, isoprene, pentanal, butyric acid, toluene, 2,5-dimethylfuran, cyclohexanone, hexanal, heptanal, acetophenone, propylcyclohexane, octanal, nonanal, decanal, 2,2-dimethyldecane	↑	0.952	89.2	89.1	89.1
Liver	Miller-Atkins et al^6^	SIFT-MS	HC *vs.* HCC	(E)-2-nonene, ethane, benzene	↑	NA	73^a^	71^a^	72
				Hydrogen sulfide	↓				
			Cirrhosis *vs.* HCC	Ethanol	↑				
				Acetone, acetaldehyde, dimethyl sulfide	↓				
	Sukaram et al^56^	GC-MS	Controls *vs.* HCC	1,4-Pentadiene, acetone, phenol, allyl methyl sulfide, d-limonene, dimethyl sulfide, hexane, 3-methyl, cyclopentane, methyl-, 1-propene, sevoflurane	↑	NA	44^b,c^	75^b,c^	55.4
				Benzene, ethylbenzene, 3,4-dimethoxycinnamic acid, methylene chloride, camphene, methyl methacrylate, furan, 2-methoxy, 4-methyl-2,4-bisp-hydroxyphenylpent-1-ene	↓				
Breast	Peng et al^55^	GC-MS	HC *vs.* BC	3,3-Dimethyl pentane	↑	NA	NA	NA	NA
				2-Amino-5-isopropyl-8-methyl-1-azulenecarbonitrile, 5-(2-methylpropyl)nonane, 2,3,4-trimethyl decane, 6-ethyl-3-octyl ester 2-trifluoromethyl benzoic acid	↓				
Colorectal	Peng et al^55^	GC-MS	HC *vs.* CRC	1,1′-(1-Butenylidene)bis benzene, 1-iodo nonane	↑	NA	NA	NA	NA
				1,3-Dimethyl benzene, [(1,1-dimethylethyl)thio] acetic acid, 4-(4-propylcyclohexyl)-4′-cyano[1,1′-biphenyl]-4-yl ester benzoic acid, 2-amino-5-isopropyl-8-methyl-1-azulenecarbonitrile	↓				
	Amal et al^57^	GC-MS	Control *vs.* CRC	Acetone, ethyl acetate	↑	NA	85	94	91
				Ethanol, 4-methyl octane	↓				
Gastric	Amal et al^58^	GC-MS	Control *vs.* GC	2-Propenenitrile, furfural, 2-butoxy-ethanol, hexadecane	↑	NA	73	98	92
	Tong et al^59^	GC-MS	Control *vs.* GC	2,3-Butanediol, [R-(R^a^,R^a^)]-, hexadecane, undecane, 3,8-dimethyl-	↑	NA	NA	NA	NA
				1,3-Dioxolan-2-one	↓				
Esophageal	Kumar et al^44^	SIFT-MS	Noncancer controls *vs.* EC	Butyric acid, pentanoic acid, hexanoic acid, phenol, methyl phenol, ethyl phenol, butanal, pentanal, hexanal, heptanal, octanal, nonanal, decanal	↑	0.9	87.5	82.9	NA
	Markar et al^5^	SIFT-MS	Controls *vs.* EC	Hexanoic acid, butanal, decanal	↑	0.85	80	81	NA
				Butyric acid, pentanoic acid	↓				
Prostate	Peng et al^55^	GC-MS	HC *vs.* PC	Toluene, 2,2-dimethyldecane	↑	NA	NA	NA	NA
				2-Amino-5-isopropyl-8-methyl-1-azulenecarbonitrile, p-xylene	↓				
Ovarian	Amal et al^60^	GC-MS	Non-cancer (BGN + TF) *vs.* OC	Decanal, 2-butanone	↑	0.84	71	71	71
Thyroid	Guo et al^61^	GC-MS	HC *vs.* PTC	Phenol, ethylene glycol mono vinyl ester, cyclopropane, 1-bromo-1-(3-methyl-1-pentenylidene)-2,2,3,3-tetramethyl	↑	1	100	100	NA
				Cyclohexanone, 4-hydroxybutyric acid, 2,2-dimethyldecane, ethylhexanol	↓				
			Nodule goiter *vs.* PTC	Cyclopentane, 1,1,3-trimethyl-3-(2-methyl-2-propenyl), trans-2-dodecen-1-ol	↑	0.9015	92	82.05	NA
				(3-Methyl-oxiran-2-yl)-methanol	↓				

^a^Data for patients with HCC *vs*. those without HCC (healthy control, cirrhosis, pulmonary hypertension, and colorectal cancer liver metastases). ^b^Data derived from the test set. ^c^Four compounds are included in the model.

AUC: Area under the curve; BC: Breast cancer; BGN: Benign genital tract neoplasia; CRC: Colorectal cancer; EC: Esophageal cancer; GC: Gastric cancer; GC-MS: Gas chromatography-mass spectrometry; HC: Healthy control; HCC: Hepatocellular carcinoma; HPPI-TOFMS: High-pressure photon ionization time-of-flight mass spectrometry; LC: Lung cancer; NA: Not applicable; OC: Ovarian cancer; PC: Prostate cancer; PTC: Papillary thyroid carcinoma; SIFT-MS: Selected ion flow tube mass spectrometry; TF: Tumor free; VOC: Volatile organic compound.

## Volatile organic compounds in treatment efficacy prediction and recurrence monitoring

### Treatment efficacy prediction

#### Systemic therapy

Exhaled breath VOCs play a crucial role in cancer detection, diagnosis, and predicting treatment efficacy. Immune checkpoint inhibitors (ICIs), a type of immunotherapy, have broad potential applications. Moreover, programmed death-ligand 1 (PD-L1) is a commonly used biomarker to enhance ICI effectiveness, albeit with moderate sensitivity and specificity. Therefore, new biomarkers are required to improve the accuracy of assessing ICI efficacy. De Vries et al^12^ conducted an initial study to determine whether the baseline exhaled breath VOC profiles could differentiate between responders and non-responders to anti-PD-1 therapy among patients with non-small cell lung cancer (NSCLC). Exhaled breath samples were collected from 143 patients with NSCLC scheduled for anti-PD-1 therapy. Patients with progressive disease (PD) were classified as non-responders, whereas those with partial response (PR) and stable disease (SD) were categorized as responders based on Response Evaluation Criteria in Solid Tumors (RECIST) Version 1.1 criteria after a 3-month follow-up. An electronic nose was used to capture VOC patterns, and a discriminant function was used to create a predictive model for distinguishing between responders and non-responders, achieving an AUC of 0.89. Buma et al^13^ investigated breath samples from 94 patients with advanced NSCLC using an electronic nose. Breath samples were collected before anti-PD-L1 therapy and 6 weeks after treatment. Patients were categorized as objective responders (PR) or non-responders (SD/PD) based on the RECIST 1.1 criteria at the 3-month follow-up. Using the model established by De Vries et al^12^ the training set achieved an AUC of 0.95, 100% sensitivity, and 73% specificity in distinguishing responders from non-responders. In the validation set, the AUC of the model was 0.97, with a sensitivity of 100% and a specificity of 76%.

Investigations into the predictive capacity of VOCs regarding anti-PD-1 therapy efficacy have demonstrated promising results. Integrating VOCs data with PD-L1 expression levels in model development is recommended, as these parameters reflect distinct aspects of the metabolic profiles of cancer patients. Utilizing both indicators in tandem may elucidate the intricacies of the host's metabolic landscape, potentially enhancing the precision of therapeutic outcome predictions and offering a more holistic framework for clinical decision-making.

Nardi-Agmon et al^11^ used an electronic nose and GC-MS to investigate the exhaled breath of 39 individuals with advanced LC, both before and 1 month after chemotherapy or targeted therapy. Three VOCs that displayed significantly reduced levels (*P* < 0.05) were identified in patients who exhibited positive responses (PR/SD) via GC-MS analysis. Exhaled breath samples were further examined using the electronic nose, and a multivariate discriminant function was used to construct a predictive model. This model demonstrated 93% sensitivity, 85% specificity, and 89% accuracy in distinguishing between patients, pre- and post-treatment. Moreover, in differentiating responders from non-responders, the model exhibited a sensitivity of 28%, a specificity of 100%, and an accuracy of 92%.

Electronic nose technology was employed as the detection method in all three studies; however, sensitivity varied across them. This variability may be attributed to several factors^62^^,^^63^: (1) patient selection criteria, including age, sex, smoking status, medication use, and gas collection environment, which considerably influence VOC profiles. (2) Discrepancies in exhaled breath collection and analysis protocols across studies, leading to divergence in outcomes. (3) Poor stability and reproducibility of the electronic nose, affecting sensor responses to gases and result reliability. (4) Technical heterogeneity among different electronic nose devices, contributing to result discrepancies. To improve the predictive consistency of electronic nose technology, future efforts should focus on refining patient selection criteria, mitigating confounding factors, standardizing collection and analysis methodologies, enhancing instrument stability, and investigating device-specific differences, thereby augmenting the reliability of electronic nose technology in clinical applications.

#### Local therapy

Sukaram et al^14^ used gas chromatography-field asymmetric ion mobility spectrometry to distinguish breath volatiles in 38 individuals diagnosed with HCC before and 1 month after undergoing TACE and ablation. Participants were divided into two groups based on their response to treatment: a response cohort and a non-response cohort, determined by the presence of residual viable tumors. Notably, a significant difference in the concentration of acetone dimers was observed between the two groups (*P* < 0.05). A predictive model was constructed by employing an XGBoost algorithm, demonstrating discriminative ability between the response and non-response cohorts, with a sensitivity of 95.7%, specificity of 73.3%, and accuracy of 86.8%. The model achieved an AUC of 0.78.

These studies highlight the potential of VOCs in predicting the therapeutic effectiveness against malignant tumors. However, compared to their use in cancer screening and diagnosis, research on VOCs for predicting therapeutic outcomes remains limited. These studies had small sample sizes, focused primarily on predicting the therapeutic effects of specific treatments, and lacked comprehensive research on predicting outcomes after combined treatments. Additionally, existing studies emphasized the predictive ability of VOCs for short-term treatment results, but lacked comprehensive investigation on their predictive role in long-term treatment effects. Therefore, further research is required to determine whether VOCs can function as predictive indicators of long-term efficacy. Prolonged patient follow-up in each study is essential to explore the potential of VOCs in predicting long-term treatment efficacy.

### Recurrence monitoring

The use of VOCs for monitoring the recurrence of malignant tumors also has promising potential. Altomare et al^64^ conducted a study using thermal-desorption gas chromatography–mass spectrometry for metabolic profiling of breath samples from 48 patients with CRC, 55 HCs, and 32 patients who underwent curative surgery without recurrence after a 1-year follow-up. This study identified 31 VOCs as significant metabolites for distinguishing between patients with CRC and the follow-up group and between the follow-up and HC groups. Of these, 11 VOCs were consistent with a previous study. A probabilistic neural network was subsequently used to develop a model based on these 11 VOCs to discriminate between the CRC and follow-up groups and between the follow-up and HC groups. The model exhibited high sensitivity and specificity in distinguishing between these groups.^23^ These findings suggest that VOC differences exist between patients with cancer and healthy individuals and between disease-free patients and healthy individuals, possibly owing to tumor-related metabolic changes. Steenhuis et al^65^ used an electronic nose to differentiate between patients with CRC without recurrence or metastasis (*n* = 36) and those with recurrence or metastasis (*n* = 26), within 5 years of radical surgery. An artificial neural network was used to establish a discrimination model with notable sensitivity and specificity. Hanevelt et al^66^ used an electronic nose to create a VOC-based monitoring tool for CRC following radical surgery. The study included 47 patients, with exhaled breath collected before and 18 days after surgery. A machine-learning algorithm was used to build the model. During the training phase, the model successfully differentiated between preoperative and postoperative CRC breath samples with a sensitivity of 78%, a specificity of 73%, and an AUC of 0.79. In the validation stage, the model demonstrated a sensitivity of 90%, specificity of 60%, and AUC of 0.82.

Three studies conducted a comparative analysis of VOCs in patients with CRC pre-surgery and post-surgery, suggesting their potential for postoperative monitoring and recurrence surveillance. However, these studies were limited by small sample sizes, necessitating larger cohorts to improve validation. Moreover, uncertainties persist regarding the uniformity of VOC distribution between patients with tumor recurrence and those with an initial tumor diagnosis. Furthermore, studies examining alterations in VOC profiles among patients following cancer metastasis are also limited.

The precise occurrence of VOC alterations following tumor treatment remains unclear, emphasizing the importance of determining optimal sampling time. Broza et al^67^ used GC-MS to identify VOC changes in patients with early LC pre- and post-surgery, finding significant modifications within 3 weeks. Wang et al^9^ used HPPI-TOFMS during the perioperative phase, noting alterations in 16 VOC concentrations 4 weeks post-surgery. Conversely, Poli et al^68^ reported that VOC levels in patients with NSCLC undergoing surgery remained stable after 1 month, except for those of isoprene. However, over a 3-year period post-surgery, specific VOC concentrations exhibited changes; pentadiene and benzene levels decreased, whereas pentane, toluene, and ethylbenzene levels increased compared to baseline. Ge et al^69^ investigated VOCs 0.5 h before and 0.5, 2, 4, and 6 h post-treatment to assess variations before and after radiotherapy. The timing of significant VOCs post-radiotherapy varied, with some exhibiting substantial changes as early as 0.5 h post-treatment, whereas others exhibited differences only after 6 h. Moreover, certain VOCs exhibited an initial increase before a subsequent decrease.

Consequently, some researchers recommend combining post-treatment breath sampling with follow-up assessments to dynamically monitor VOC changes in patients.^68^ Further validation is required to determine whether post-treatment VOC alterations are associated with treatment modalities.

This review has some limitations. First, it provided a cursory introduction to VOC detection equipment, lacking an exhaustive delve into the specifics of each device. Second, the focus of this review was detecting VOCs in prevalent solid tumors, excluding hematological malignancies and neurologic tumors.

## Conclusions

VOC detection in exhaled breath offers several advantages, such as non-invasiveness, convenience, and high accuracy, making it valuable for diagnosing malignant tumors, predicting therapy outcomes, and monitoring post-treatment progress. However, further investigation is needed to comprehensively understand the mechanisms behind VOC production, variations in VOC levels among patients with cancer, and changes in VOC profiles following treatment. Moreover, the existence of tumor-specific VOCs remains unconfirmed, necessitating further research into the molecular processes governing VOC production and changes. Efforts are also crucial to distinguish between VOC distributions in patients with malignant tumors and healthy individuals, identify VOCs associated with various tumor types, and assess potential changes in VOC profiles at different stages. Improving the accuracy and reliability of detection equipment, standardizing VOC detection procedures, and establishing detection criteria are critical for enhancing VOC analysis. Based on the latest research, the initial use of GC-MS for qualitative and quantitative analyses of VOCs in patients with cancer is recommended to establish a specific VOC profile for different tumor types. Subsequently, electronic nose technology should be applied to detect and model complex VOC mixtures, providing a more robust foundation for clinical tumor screening, diagnosis, and prognosis.

## Authors contribution

Conceptualization, Mian Xi; writing—original draft preparation, Yilu Gao; writing—review and editing, Baoqing Chen, Xingyuan Cheng, Shiliang Liu, Qiaoqiao Li, and Mian Xi. All the authors have read and approved the final version of the manuscript.

## Ethics statement

None.

## Declaration of generative AI and AI-assisted technologies in the writing process

The authors declare that generative artificial intelligence (AI) and AI assisted technologies were not used in the writing process or any other process during the preparation of this manuscript.

## Funding

None.

## Data availability statement

The datasets used in the current study are available from the corresponding author on reasonable request.

## Conflict of interest

The authors declare that they have no known competing financial interests or personal relationships that could have appeared to influence the work reported in this paper.
